# Nutrient-Dependent Impact of Microbes on *Drosophila suzukii* Development

**DOI:** 10.1128/mBio.02199-17

**Published:** 2018-03-20

**Authors:** XiaoLi Bing, Joseph Gerlach, Gregory Loeb, Nicolas Buchon

**Affiliations:** a Department of Entomology, Cornell University, Ithaca, New York, USA; b Department of Entomology, Cornell University, Geneva, New York, USA; University of Hawaii at Manoa

**Keywords:** *Drosophila suzukii*, TOR, development, fruit, insulin pathway, larval growth, microbiota, protein starvation

## Abstract

*Drosophila suzukii* Matsumura is an invasive species of vinegar fly that has become a prominent pest of berries and other soft-skinned fruits. Unlike most other *Drosophila* species, female *D. suzukii* flies lay their eggs in ripening and ripe fruits and larvae develop within the fruit. To understand how *D. suzukii* larvae utilize ripe and ripening fruits, which usually have low levels of protein, we investigated the microbiota of field-captured and laboratory-reared *D. suzukii* flies and further examined the combined influence of diet and microbes on host fitness. Field-captured flies were associated with diverse microbiota, which varied significantly with sampling location and season. In contrast, laboratory-reared flies possessed strikingly lower bacterial abundance and diversity. A comparison of conventionally reared (CR) and germ-free (GF) flies revealed that the microbiota of *D. suzukii* does not alter its development significantly but decreases its life span under conditions of a nutrient-sufficient diet. However, the microbiota is essential for *D. suzukii* development on strawberry-based or blueberry-based fruit diets. This developmental failure could be rescued by reassociation with single bacterial or fungal species or by the addition of a high quantity of heat-killed microbes. In addition, we found that proteins are limiting with respect to fly development on fruit-based diets and that GF flies show signs of protein starvation. Taken together, our study results demonstrate that the microbiota provides key proteins required for the development of *D. suzukii* reared on fresh fruit. Our work shows that the impact of microbes on fly fitness depends strongly on nutritional conditions.

## IMPORTANCE

Animals are commonly associated with specific microbes, which play important roles in host development and fitness. However, little information about the function of microbes has been available for the important invasive pest *Drosophila suzukii*, also known as Spotted wing drosophila. Our study results demonstrate that the abundance and structure of microbiota in *D. suzukii* are strongly affected by the environment, where microbes have variable roles depending on the nutritional situation. For instance, we found that the presence of microbes is deleterious for flies growing on a protein-rich diet and yet is beneficial for flies growing on a diet of protein-poor fruits. Additionally, germ-free flies must feed on microbes to obtain the necessary protein for larval development on strawberries and blueberries. Our report validates the complexity seen in host-microbe interactions and may provide information useful for *D. suzukii* pest control.

## INTRODUCTION

The influence of microbes on host physiology is known in a broad range of animals, including insects. Microbial activities in insects range from pathogenic to beneficial. Although earlier studies of insect-microbe interactions had emphasized the relationships between pathogens and insects (1–3), recent research has also shown the importance of microbes for host development and physiology (4–6). In insects that feed on nutrient-restricted diets (e.g., plant xylem, plant phloem sap, animal blood), microbes supply key nutrients that are lacking from the diet and are essential for normal host development (7–11). For example, the intracellular gut bacterium *Wigglesworthia glossinidia* provides essential nutrition (e.g., B vitamins) to its insect host, the tsetse fly, and is necessary for maintaining fly fecundity and development of larval immunity (12, 13). Additionally, microbes have been reported as influential factors of biological pest invasion (14).

Recently, *Drosophila melanogaster* has emerged as a model to study the impact of associated microbes (15–17). *D. melanogaster* is commonly associated with a low number of bacterial species, i.e., 5 to 10 in the laboratory, mostly composed of *Acetobacter* and lactobacilli (18). The impact of *D. melanogaster* microbiota on its host is complex, with both beneficial and deleterious effects on host physiology. *D. melanogaster*-associated microbes have been shown to promote larval development and alter host metabolism (16, 17, 19) but also to negatively impact host longevity (20–23). Additionally, these microbes strongly alter intestinal physiology, promoting basal turnover and altering intestinal stem cell lineage (20, 21, 24). Interestingly, the complexity of the effects that *D. melanogaster*-associated microbes have on host physiology and the requirement of specific bacterial species seem to depend on diet. For instance, *Lactobacillus plantarum* is able to promote larval growth in a target of rapamycin (TOR)-dependent manner, while *Acetobacter pomorum* promotes larval growth on food depleted of amino acids in an insulin pathway-dependent manner (16, 17). However, it remains unclear what the role of gut microbes is in other *Drosophila* species and especially under conditions relevant to their life history and ecosystem.

*Drosophila suzukii* Matsumura (Diptera: Drosophilidae), or spotted wing drosophila, is a new invasive pest of berries and other soft-skinned fruits that has spread quickly from its native continent of Asia to the Americas and Europe (25). With a serrated ovipositor, the female *D. suzukii* is capable of oviposition directly into intact ripe or ripening fruit (26). As a result, the larvae of *D. suzukii* feed and grow in fresh fruit, in contrast to the overripe and rotting fruits that the larvae of most other fruit-infesting *Drosophila* utilize (27). The fruits infested by *D. suzukii* are subsequently rendered unmarketable, thereby causing substantial economic losses to the fruit industry (28). Fruits consist mostly of sugar rather than protein, and ripening fruits are especially nutritionally unbalanced. The role of microbes in allowing *D. suzukii* flies to complete their development on fruits is an important and yet unexplored issue.

Moreover, the fact that *D. suzukii* specializes on ripe and ripening fruit (29), which makes it a significant economic pest, implies that studying associated microbes and their impact on *D. suzukii* is key to understanding the mechanisms of biological invasion and may provide information for biological pest control. Although a few studies investigating microbe diversity in *D. suzukii* have recently been reported (30–33), few have focused on the impact of associated microbes on the development and survival of *D. suzukii* (34, 35).

In this study, we identified the microbes associated with wild-caught and laboratory-reared *D. suzukii*. We found that *D. suzukii* is associated with a high diversity of microbes in the wild and that microbiota composition varies largely by location and season. Interestingly, we found a restricted bacterial load in flies reared under laboratory conditions, suggesting that the *D. suzukii* microbiota is mostly composed of dietary microbes. We next characterized the effect of this microbiota on fly development, fitness, and survival under laboratory conditions. We found that, overall, germ-free (GF) flies perform similarly to or better than their conventionally reared (CR) counterparts on a nutrient-sufficient diet, suggesting that those microbes have slightly deleterious effects on host physiology under those conditions. However, GF flies were unable to successfully develop on two different fruit-based diets, whereas CR flies could, suggesting a key requirement for associated microbes in *D. suzukii* development under conditions similar to those encountered in the wild. This growth-promoting effect was not species specific, as multiple microbial species were able to promote growth. GF larvae raised on a fruit-based diet showed symptoms of protein starvation. Development could be rescued by the addition of proteins to the diet, suggesting that these microbes provide *D. suzukii* with key proteins. Overall, our analysis shows that microbes play various roles in *D. suzukii* and that those roles largely depend on the environment and on the available nutrient resources.

## RESULTS

### Bacterial diversity of *D. suzukii* is significantly affected by habitat.

We first aimed to identify the microbes commonly associated with *D. suzukii* under wild-type conditions as well as in laboratory stocks using both culture-dependent (colony identification on De Man, Rogosa, and Sharpe [MRS], lysogeny broth [LB], and yeast extract-malt extract-peptone-dextrose agar [SDAY] plates) and culture-independent (16S ribosomal DNA [rDNA] sequencing) methods.

**FIG 1:**
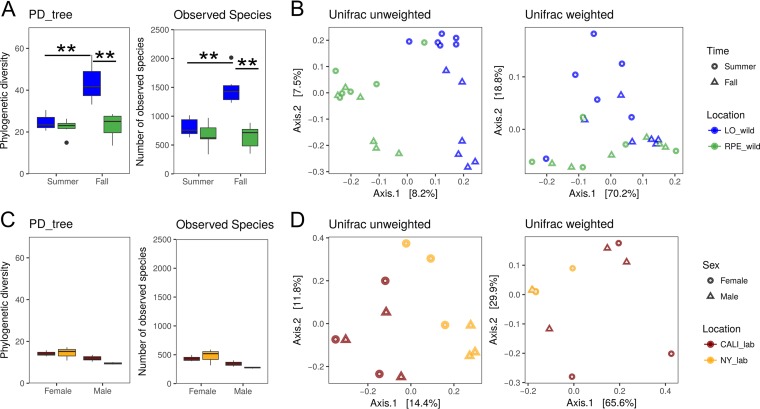
Bacterial community variation among wild and laboratory *D. suzukii* flies. (A) Box plots of phylogenetic diversity (PD_tree) and number of observed species of microbiota from wild *D. suzukii* flies. (For PD data, location *P* = 0.0006, time *P* < 0.0001, and sex *P* = 0.0050; for observed-species data, location *P* = 0.0002, time *P* = <0.0001, and sex *P* = 0.0103). (B) Unweighted and weighted UniFrac-based principal-component analysis (PCoA) plots of bacterial communities in wild *D. suzukii* flies. (For unweighted data, location *P* = 0.001, time *P* = 0.001, and sex *P* = 0.149; for weighted data, location *P* = 0.095, time *P* = 0.03, and sex *P* = 0.196). (C) Box plots of phylogenetic diversity and number of observed species of microbiota from laboratory *D. suzukii* flies. (For PD data, location *P* = 0.0578 and sex *P* = 0.0128; for observed-species data, location *P* = 0.188 and sex *P* = 0.014). (D) Unweighted and weighted UniFrac-based PCoA plots of bacterial communities in laboratory *D. suzukii* flies. (For unweighted data, location *P* = 0.005 and sex *P* = 0.128; for weighted data, location *P* = 0.015 and sex *P* = 0.321). Statistically significant differences of alpha diversities were analyzed using three-way ANOVA with Tukey *post hoc* tests. Statistically significant differences in beta diversities were analyzed using Adonis analysis with 999 Monte Carlo permutations. **, *P* < 0.01.

We began by characterizing the abundance and diversity of gut microbes that are associated with *D. suzukii* in the wild. In order to determine the impact of the environment on bacterial composition, flies were collected in two seasons (summer and fall) from two distinct locations (fruit farms represented here by the abbreviations LO and RPE) in Geneva, NY. Levels of bacterial alpha diversity were significantly different among flies sampled at different collection locations, in different seasons, and by sex (Fig. 1A; see also Fig. S1A and C and Table S1 in the supplemental material). This suggests that the environment plays a major role in determining which microbes are associated with *D. suzukii*. Microbes were generally more abundant in number and more diverse in flies collected from LO than in those collected from RPE (Fig. 1A; see also Fig. S1A). Flies also harbored more bacteria in fall than in summer (Fig. 1A; see also Fig. S1C). The bacterial community structures were quantitatively different between sampling locations (Fig. 1B; see also Fig. S1B) and both qualitatively and quantitatively different between collecting seasons (Fig. 1B; see also Fig. S1D). Although the abundance (numbers and diversity of bacterial species) of microbes was slightly greater in female flies than in male flies, no significant difference in bacterial beta diversity was observed between sexes (Fig. S1E and F; see also Table S1), indicating that sex affects only the number of bacteria associated with *D. suzukii*. Collectively, our data indicate that *D. suzukii* harbors various microbial populations in different environmental habitats, suggesting an inconstant host microbiota, as has been reported for other *Drosophila* species (36, 37).

10.1128/mBio.02199-17.1 FIG S1  Bacterial community variation among wild and laboratory *D. suzukii* flies. (A, C, and E) Box plots of phylogenetic diversity and number of observed species of microbiota from wild *D. suzukii* flies classified by (A) sampling location, (C) sampling time, and (E) fly sex. (Statistical significance levels are shown in plot figures.) (B, D, and F) Unweighted and weighted UniFrac-based PCoA plots of bacterial communities in wild *D. suzukii* flies classified by (B) sampling locations, (D) collection time, and (F) fly sex. (Statistical significance levels are shown in plot figures.) (G and I) Box plots of phylogenetic diversity and number of observed species of microbiota from wild *D. suzukii* flies classified by (G) sampling location and (I) fly sex. (Statistical significance levels are shown in plot figures.) (H and J) Unweighted and weighted UniFrac-based PCoA plots of bacterial communities in wild *D. suzukii* flies classified by (H) sampling locations and (J) fly sex. The statistical significance of differences of alpha diversities was analyzed using Student’s *t* test. The statistical significance of differences in beta diversities was analyzed using Adonis analysis with 999 Monte Carlo permutations. Statistical significance levels are shown in plot figures. *, *P* < 0.05. **, *P* < 0.01. NS, not statistically significant. Download FIG S1, TIF file, 1.5 MB. Copyright © 2018 Bing et al. 2018 Bing et al. This content is distributed under the terms of the Creative Commons Attribution 4.0 International license.

10.1128/mBio.02199-17.6 TABLE S1  Detailed data and statistics from analysis of microbially diverse populations. Download TABLE S1, XLSX file, 0.04 MB. Copyright © 2018 Bing et al. 2018 Bing et al. This content is distributed under the terms of the Creative Commons Attribution 4.0 International license.

**FIG 2:**
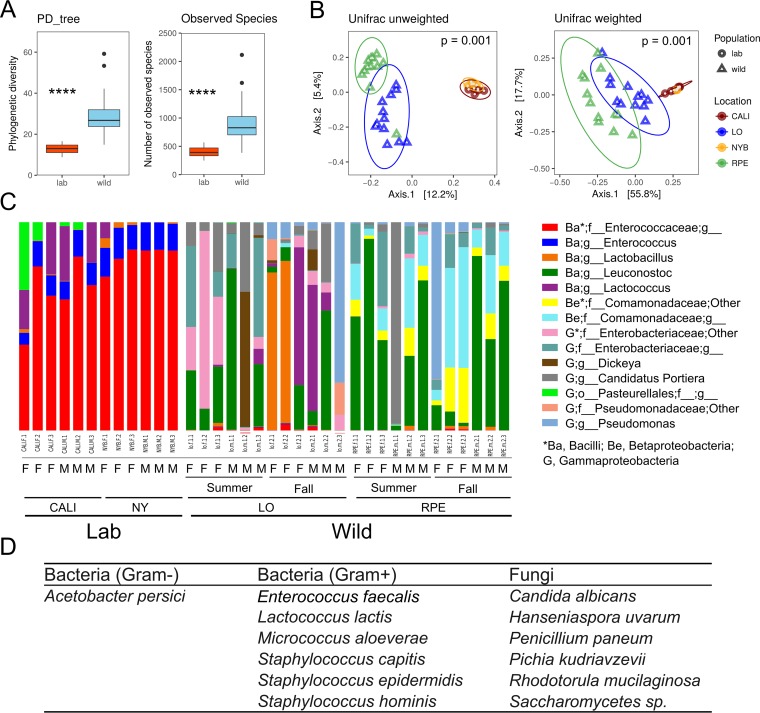
The diversity of microbes in wild *D. suzukii* flies is much higher than in laboratory flies. (A) Box plots of phylogenetic diversity (PD tree) and number of observed species of microbiota from laboratory and wild *D. suzukii* flies. (B) Unweighted and weighted UniFrac-based PCoA plots of bacterial communities in laboratory and wild *D. suzukii* flies. Statistically significant differences of alpha diversities were analyzed using the Wilcoxon rank sum test. Statistically significant differences in beta diversities were analyzed using Adonis analysis with 999 Monte Carlo permutations. Statistical significance levels are shown in plot figures. ****, *P* < 0.0001. (C) Taxonomic composition of microbial communities associated with *D. suzukii* flies. Each bar is indicated by a different color at the genus level (>0.5% of average relative abundance in groups). F, female; M, male. (D) List of isolated culture-dependent microbes from laboratory *D. suzukii* flies.

We next focused on two laboratory stocks that were originally collected from distinct wild populations and that have been reared on the same diet in the laboratory for more than 10 generations. The bacterial communities in the two laboratory stocks were found to have comparable levels of abundance (Fig. 1C; see also Fig. S1G and Table S1) but were different in their microbial composition (Fig. 1D; see also Fig. S1H and Table S1). Similarly to the trend found in wild flies, laboratory female flies had slightly more bacteria than male flies (Fig. S1I), but similar microbial population structures were found in the two groups (Fig. 1D; see also Fig. S1J). Strikingly, both bacterial abundance and diversity were strongly reduced in laboratory-reared stocks compared to wild-caught flies. Analysis of alpha diversity revealed that bacterial abundance and diversity were significantly higher in wild flies than in laboratory flies (Fig. 2A). The unweighted and weighted UniFrac distances depicted on principal-component-analysis (PCA) plots further supported this conclusion (Fig. 2B). Taken together, our results suggest that being raised on a uniform, laboratory diet significantly decreased microbially diverse populations of flies but still allowed them to maintain some of their particular microbial patterns.

We next looked at the composition of the bacterial communities of wild-caught and laboratory-raised flies. The most abundant bacteria detected in wild *D. suzukii* flies were *Lactobacillus*, *Leuconostoc*, *Lactococcus*, *Comamonadaceae*, and *Enterobacteriaceae* (Fig. 2C), which were also prevalent in other *Drosophila* surveys (32, 37, 38). In laboratory-raised flies, the most abundant bacteria are *Enterococcaceae* (39), which corresponded to roughly 87% of the sequencing reads (Fig. 2C). In parallel to this sequence-based strategy, we identified the main bacteria associated with laboratory-raised *D. suzukii* by culture-dependent methods. Specifically, we crushed laboratory flies in phosphate-buffered saline (PBS) solution and plated the suspension on LB, MRS, and SDAY plates, allowing us to recover both bacteria and fungi/yeasts. On the basis of colony morphology as well as Sanger sequencing of isolated colonies, we identified 1 species of Gram-negative bacterium, 6 species of Gram-positive bacteria, and 6 species of fungi (Fig. 2D; see also Table S1). The finding of *Enterococcus faecalis* and *Lactococcus lactis* confirmed the finding of the presence of these bacteria in the sequencing results (Fig. 2D). We additionally cultured *Acetobacter* and *Staphylococcus* from flies; those species were found in only a small proportion of our sequencing data (Table S1). Five of the 6 identified fungal species were yeast species, including *Candida*, *Hanseniaspora*, *Pichia*, *Rhodotorula*, and *Saccharomycetes* species, several of which are reported to be frequently associated with *Drosophila* (30, 40, 41). Taken together, our results demonstrate that *D. suzukii* harbors a wide variety of microbes in the wild and that some, but not all, of these microbes are able to remain associated in laboratory settings.

### Microbiota presence is deleterious or neutral for *D. suzukii* on a nutrient-sufficient diet.

*D. suzukii* larvae, in contrast to most drosophilids, develop in fresh fruits and are therefore assumed to eat a microbe-poor fruit diet (27). We next decided to investigate the role of the microbes associated with *D. suzukii* in its development, fitness, and physiology. As we had demonstrated that *D. suzukii* laboratory stocks are associated with a lower diversity of microbes, we first tested whether the microbes found in our stocks were strongly pathogenic to *D. suzukii*. To this purpose, we performed oral infection of *D. suzukii* by the use of laboratory-isolated bacteria or fungi and tracked the survival of the flies. None of the microbes that we isolated strongly affected fly survival (<25% mortality at 7 days after infection) (Fig. S2), suggesting that we had not isolated major pathogens in our laboratory microbial community.

10.1128/mBio.02199-17.2 FIG S2  None of cultured microbes from laboratory *D. suzukii* flies were orally pathogenic to *D. suzukii*. (A and B) Survival curves of *D. suzukii* adult flies after feeding with laboratory-isolated bacteria and fungi at the following two doses: (A) OD_600_ = 10 and (B) OD_600_ = 200. The curves represent average percent survival (*n* ≥ 60). More than 75% of the tested flies were alive on all microbes at 7 days after feeding. Download FIG S2, TIF file, 0.5 MB. Copyright © 2018 Bing et al. 2018 Bing et al. This content is distributed under the terms of the Creative Commons Attribution 4.0 International license.

**FIG 3:**
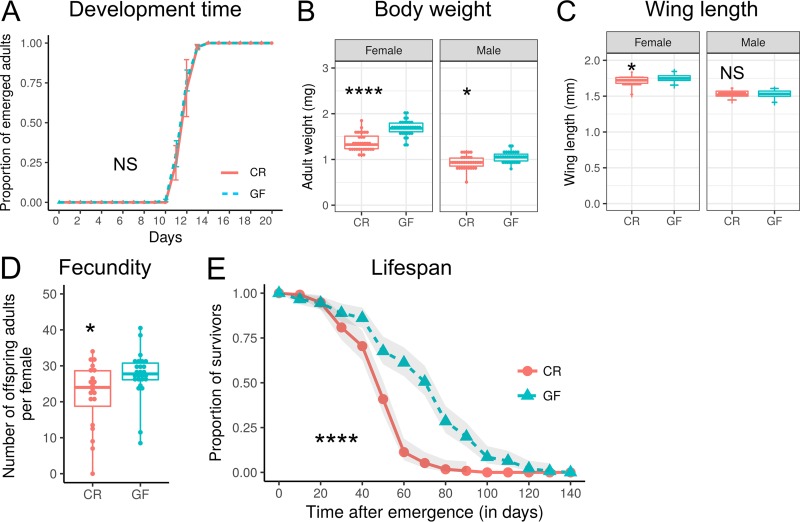
Microbes are deleterious to *D. suzukii* flies on a nutrient-rich sucrose-yeast diet. (A) Developmental time of CR and GF flies growing on a sucrose-yeast diet. The cumulative percentage of the adult emergence time is shown over time. Data represent means ± standard errors of the means (SEM). (For vials, CR *n* = 6 and GF *n* = 9; for individuals, CR *n* = 157 and GF *n* = 183). (B) Body weights of newly emerged CR and GF adult flies. (CR and GF pairs for both sexes, *n* = 32 to 33). (C) Wing lengths of newly emerged CR and GF adult flies. (CR and GF flies for both sexes, *n* = 53 to 65). (D) Fecundity of CR and GF adult flies. (CR, *n* = 20; GF, *n* = 26). (E) Life span curve of CR and GF adult flies on a sucrose-yeast diet. (CR, *n* = 121; GF, *n* = 146). The curves represent average percent survival, and gray ribbons represent the 95% confidence interval. Means of emergence times, body weights, wing lengths, and numbers of offspring were compared using Student’s *t* test or the Wilcoxon rank sum test. Developmental and life span curves were analyzed with the Cox proportional-hazards model test. (*, *P* < 0.05; **, *P* < 0.01; ****, *P* < 0.0001; NS, not statistically significant.)

We next aimed to study the impact of the microbiota on *D. suzukii* development and fitness by generating GF *D. suzukii* flies through egg surface sterilization. Afterward, we compared the data corresponding to body size, fecundity, life span, and the timing of adult progeny emergence in the absence (GF) and presence (CR) of microbiota on a nutrient-rich sucrose-yeast diet that contained 4% sucrose, 5% yeast, and 6% yellow cornmeal. The findings with respect to time to emergence were similar for CR and GF flies (the average emergence time was ~11.94 days in GF flies versus ~12.19 days in CR flies) (Fig. 3A), showing that microbes are not essential for *D. suzukii* development on a sucrose-yeast diet. The newly emerged GF adults were significantly heavier (the average body weights per fly were 1.69 mg for females and 1.05 mg for males in GF flies versus 1.37 mg for females and 0.94 mg for males in CR flies), and GF females were larger than the control CR females (the average wing length was 1.75 mm in GF flies versus 1.72 mm in CR flies), as indicated by their larger wing size (Fig. 3B and C), suggesting that the microbiota limited larval growth to some extent. In addition, significantly more adult offspring from the first-generation flies emerged from the tubes containing eggs laid by GF flies than from the tube of CR flies (Fig. 3D). The life span of *D. suzukii* GF adults was also much longer than that of CR adults (the median survival time was 71 days in GF versus 47 days in CR) (Fig. 3E). Taken together, our results demonstrate that the *D. suzukii* microbiota is not essential for survival or development on sucrose-yeast food and that microbes act as a small but significant burden that limits host growth and life span.

### Microbiota is required for *D. suzukii* development on a protein-poor, fruit-based diet.

**FIG 4:**
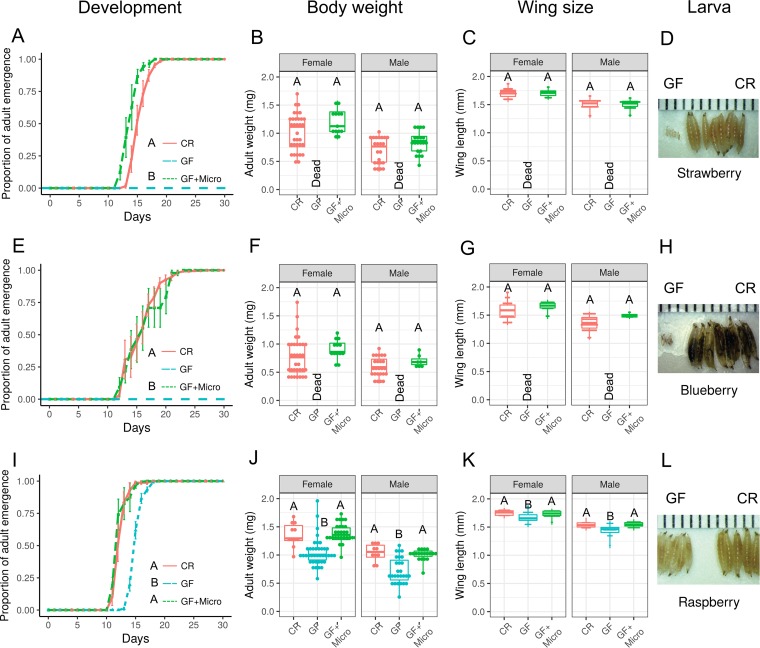
Microbes are beneficial to *D. suzukii* on a nutrient-poor diet. (A, E, and I) Developmental time of CR, GF, and GF+ Micro (microbiota-associated) flies on strawberry (A), blueberry (E), and raspberry (I). The cumulative percentage of the adult emergence time is shown over time. Data represent means ± SEM. (B, F, and J) Body weights of newly emerged CR, GF, and GF+ Micro adult flies on strawberry (B), blueberry (F), and raspberry (J). (C, G, and K) Wing lengths of newly emerged CR, GF, and GF+ Micro adult flies on strawberry (C), blueberry (G), and raspberry (K). (D, H, and L) The sizes of larvae at 5 days of development after egg deposition on strawberry (D), blueberry (H), and raspberry (L). Spaces between two adjacent black lines on the ruler represent 1 mm. Developmental curves were analyzed with the Cox proportional-hazards model test. Data were analyzed using Student’s *t* test or ANOVA followed by Tukey *post hoc* tests or the Wilcoxon rank sum test or Kruskal-Wallis test followed by Dunn’s test with Bonferroni correction depending on the results of a Shapiro-Wilk normality test. Different capital letters indicate significant statistical difference at a *P* value of <0.01. "Dead" indicates that no adult fly survived that treatment. Values equaling zero were excluded from statistical analysis. (The total numbers of the replicates are shown in Table S2.)

While the laboratory sucrose-yeast diet was rich in protein (~2% protein [42]), *D. suzukii* naturally feeds on ripe and ripening fruits that are high in sugar but low in protein. To further characterize the roles of *D. suzukii* microbiota in a more natural setting, we next assessed the development of GF and CR flies on a fruit-based diet consisting of a jellified solution of ripe fruits (strawberry, blueberry, or raspberry). GF larvae failed to develop into adults on both strawberry- and blueberry-based diets (Fig. 4A and E), while CR larvae emerged around 10 to 13 days after egg deposition on these diets. At 5 days after egg deposition, most GF larvae were still in the first instar stage. Under these conditions, GF larvae were ~1/5 the size of CR larvae collected at the same time after egg deposition (Fig. 4D and H). GF flies were able to emerge as adults on raspberry, but their emergence time was significantly delayed (~3-day lag behind CR flies) (Fig. 4I), and the merged adults were much smaller (Fig. 4J to L). The development of GF larvae was rescued to levels similar to or higher than those seen with CR larvae when we reintroduced the microbiota of CR flies to the GF fruit-based diet (Fig. 4A, E, and I), demonstrating the key role of the gut microbiota in fly development. Reintroduction of microbes led to the emergence of flies with body size and wing length comparable to those of CR adult flies (Fig. 4B and C, F and G, and J and K). Our results demonstrate that the *D. suzukii* microbiota is required for fly development on a fruit-based diet. We next wondered whether this was specific to *D. suzukii* or whether a similar dependence with respect to the microbiota would be seen with another *Drosophila* species, such as *D. melanogaster*, which develops on rotten fruit. We found that *D. melanogaster* microbes were also required for fly development on our fruit-based diets (Fig. S3). Taken together, the results presented above show that the microbiota is essential for the development of multiple *Drosophila* species on fruit-based diets.

10.1128/mBio.02199-17.3 FIG S3  Microbes are beneficial to *D. melanogaster* on a nutrient-poor diet. (A, E, and I) Developmental time of CR, GF, and GF+ Micro (microbiota-associated) flies on strawberry (A), blueberry (E), and raspberry (I). Data representing the cumulative percentage of the adult emergence time compared to the total time are shown. Data represent means ± SEM. (B, F, and J) Body weights of newly emerged CR, GF, and GF+ Micro adult flies on strawberry (B), blueberry (F), and raspberry (J). (C, G, and K) Wing lengths of newly emerged CR, GF, and GF+ Micro adult flies on strawberry (C), blueberry (G), and raspberry (K). (D, H, and L) The sizes of larvae at 5 days of development after egg deposition on strawberry (D), blueberry (H), and raspberry (L). Spaces between two adjacent black lines on the ruler represent 1 mm. Developmental curves were analyzed with the Cox proportional-hazards model test. Data were analyzed using Student’s *t* test or ANOVA followed by Tukey *post hoc* tests or the Wilcoxon rank sum test or Kruskal-Wallis test followed by Dunn’s test with Bonferroni correction depending on the results of a Shapiro-Wilk normality test. Different capital letters indicate significant statistical difference at a *P* value of <0.01. “Dead” indicates that no adult fly survived that treatment. Values equaling zero were excluded from statistical analysis. (The total numbers of the replicates are shown in Table S2.) Download FIG S3, TIF file, 2.5 MB. Copyright © 2018 Bing et al. 2018 Bing et al. This content is distributed under the terms of the Creative Commons Attribution 4.0 International license.

10.1128/mBio.02199-17.7 TABLE S2  Detailed data from analysis of microbial function. Download TABLE S2, XLSX file, 0.03 MB. Copyright © 2018 Bing et al. 2018 Bing et al. This content is distributed under the terms of the Creative Commons Attribution 4.0 International license.

### The development-promoting effect of the microbiota is not species specific.

**FIG 5:**
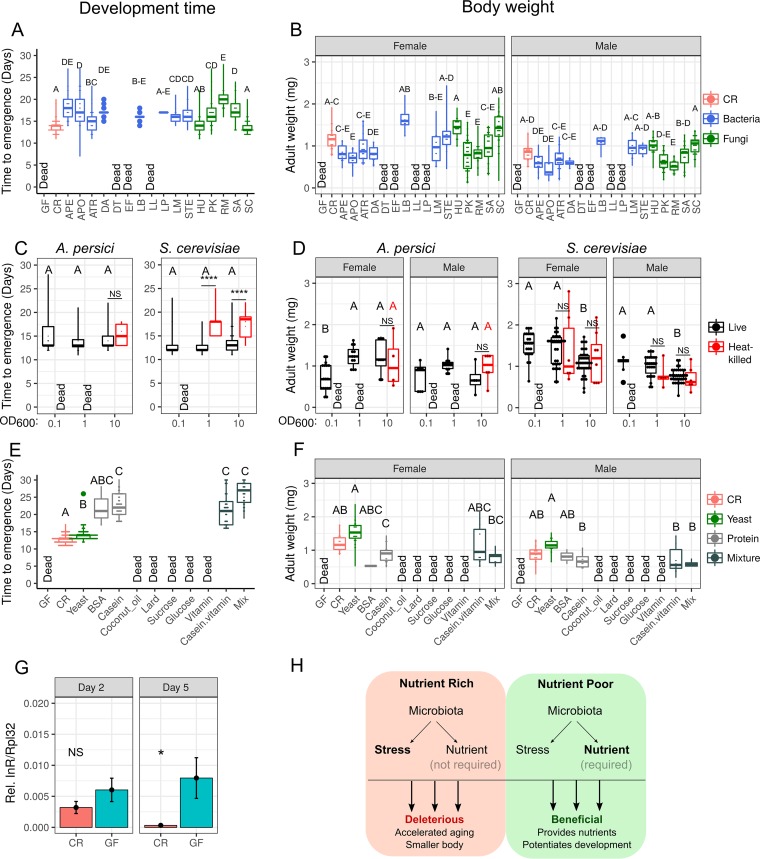
Multiple microbes provide protein for *D. suzukii* to finish development on a strawberry diet. (A and B) Time to emergence (A) and body weights (B) of flies associated with bacteria (blue) and fungi (green). APE, *A. persici*; APO, *A. pomorum*; ATR, *A. tropicalis*; DA, *D. acidovorans*; DT, *D. tsuruhatensis*; EF, *E. faecalis*; LB, *L. brevis*; LP, *L. plantarum*; LL, *L. lactis*; LM, *L. mesenteroides*; STE, *S. epidermidis*; HU, *H. uvarum*; PK, *P. kudriavzevii*; RM, *R. mucilaginosa*; SA, *Saccharomycetes* sp.; SC, *S. cerevisiae*. (C and D) Time to emergence (C) and body weights (D) of flies associated with different doses of live (black) and heat-killed (red) *A. persici* and *S. cerevisiae*. (E and F) Time to emergence (E) and body weights (F) of flies on a strawberry diet supplemented with protein (gray) or with lipid or sugar or with a mixture of lipid and sugar (dark gray). Data were analyzed using Student’s *t* test or ANOVA followed by Tukey *post hoc* tests or the Wilcoxon rank sum test or Kruskal-Wallis test followed by Dunn’s test with Bonferroni correction depending on the results of a Shapiro-Wilk normality test. Different capital letters indicate significant statistical difference at a *P* value of <0.01. "Dead" indicates that no adult fly survived that treatment. Values equaling zero were excluded from statistical analysis. (The total numbers of the replicates are shown in Table S2.) (G) Relative levels of gene expression of *InR* in CR (red) and GF (green) larvae that were grown on a strawberry diet for 2 days and 5 days after egg deposition. Relative Δ*_CT_*^*InR*^/Δ_*CT*_^*Rpl32*^ ratios are represented. Data represent means ± SEM. *n* = 8 to 9. NS, not statistically significant. *, *P* < 0.05 (Wilcoxon rank sum test). (H) Model of the roles of microbes in *D. suzukii* under conditions of nutritional availability. Microbes are not required and are even deleterious to *D. suzukii* under nutrient-rich conditions, resulting in a slightly lower developmental rate, a smaller body, and accelerated aging. Microbes are required for and beneficial to *D. suzukii* growth under nutrient-poor conditions, helping larvae to grow and complete development.

We next aimed to establish whether only specific members of the microbiota are responsible for sustaining *D. suzukii* development on fruit-based diets or if the presence of most of the members is sufficient for larval development on fruit. We selected representatives of abundant microbes obtained from our bacterial 16S rRNA gene sequencing results (*Delftia*, *Enterococcus*, *Lactobacillus*, *Lactococcus*, and *Leuconostoc*) as well as bacteria and fungi identified from our laboratory stocks (*Acetobacter*, *Staphylococcus*, *Hanseniaspora*, *Pichia*, *Rhodotorula*, and *Saccharomycetes*) and reassociated these microbes with GF *D. suzukii* to observe the effect of single microbial species on larval developmental rate and body size (Fig. 5; see also Fig. S4 and S5). Reassociation of *D. suzukii* with many of our single bacterial species rescued the development of GF flies, with different bacteria rescuing developmental rate and body size to various extents (Fig. 5A and B; see also Fig. S4A and B and S5A and B). Furthermore, a few bacterial species failed to rescue development of flies on fruit-based diets (e.g., *Delftia tsuruhatensis*, *E. faecalis*, and *L. lactis* failed to rescue development of flies on a strawberry-based diet and *Delftia acidovorans* and *L. mesenteroides* failed to rescue development of flies on a blueberry-based diet) (Fig. 5A; see also Fig. S5A), suggesting that not all bacteria associated with *D. suzukii* can sustain its development. In contrast, the five tested fungal species all successfully rescued *D. suzukii* development on both fruit-based diets. Additionally, flies that were monoassociated with *Hanseniaspora* or *Saccharomyces* had developmental rates, body sizes, and wing lengths similar to those of CR flies (Fig. 5A and B; see also Fig. S4A and B and S5A and B). Finally, we reassociated *D. suzukii* with both a bacterium (*Acetobacter persici*) and a fungus (*S. cerevisiae*) in a dual-reassociation treatment and found the rescuing efficiency to be intermediate compared to the rescue efficiency obtained with the bacterium or the fungus alone (Fig. S4C and D), which suggests that yeast-*Acetobacter* interaction, which has been reported to alter host behavior (43), is not central to *D. suzukii* growth. In sum, the results presented above suggest that most microbial species can sustain development of *D. suzukii* on a fruit-based diet but that the beneficial effects of each microbe can vary.

10.1128/mBio.02199-17.4 FIG S4  Multiple microbes help *D. suzukii* to finish development on fruit diets. (A) Wing lengths of bacterium (blue)- or fungus (green)-monoassociated flies on a strawberry diet. (B) Wing lengths of bacterium (blue)- or fungus (green)-monoassociated flies on a blueberry diet. APE, *A. persici*; APO, *A. pomorum*; ATR, *A. tropicalis*; DA, *D. acidovorans*; DT, *D. tsuruhatensis*; EF, *E. faecalis*; LB, *L. brevis*; LP, *L. plantarum*; LL, *L. lactis*; LM, *L. mesenteroides*; STE, *S. epidermidis*; HU, *H. uvarum*; PK, *P. kudriavzevii*; RM, *R. mucilaginosa*; SA, *Saccharomycetes* sp.; SC, *S. cerevisiae*. (C and D) Time to emergence (C) and body weights (D) of *A. persici-* and *S. cerevisiae*-monoassociated and dually associated flies on a strawberry and blueberry diet. APE, *A. persici*, SC, *S. cerevisiae*, APE + SC, *A. persici* and *S. cerevisiae*. Data were analyzed using Student’s *t* test or ANOVA followed by Tukey *post hoc* tests or the Wilcoxon rank sum test or Kruskal-Wallis test followed by Dunn’s test with Bonferroni correction depending on the results of a Shapiro-Wilk normality test. Different capital letters indicate significant statistical difference at a *P* value of <0.01. "Dead" indicates that no adult fly survived that treatment. Values equaling zero were excluded from statistical analysis. (The total numbers of the replicates are shown in Table S2.) Download FIG S4, TIF file, 1.1 MB. Copyright © 2018 Bing et al. 2018 Bing et al. This content is distributed under the terms of the Creative Commons Attribution 4.0 International license.

10.1128/mBio.02199-17.5 FIG S5  Multiple microbes provide protein for *D. suzukii* to finish development on a blueberry diet. (A and B) Time to emergence (A) and body weights (B) of flies associated with bacteria (blue) and fungi (green). APE, *A. persici*; APO, *A. pomorum*; ATR, *A. tropicalis*; DA, *D. acidovorans*; DT, *D. tsuruhatensis*; EF, *E. faecalis*; LB, *L. brevis*; LP, *L. plantarum*; LL, *L. lactis*; LM, *L. mesenteroides*; STE, *S. epidermidis*; HU, *H. uvarum*; PK, *P. kudriavzevii*; RM, *R. mucilaginosa*; SA, *Saccharomycetes* sp.; SC, *S. cerevisiae*. (C and D) Time to emergence (C) and body weights (D) of flies associated with different doses of live (black) and heat-killed (red) *A. persici* and *S. cerevisiae*. (E and F) Time to emergence (E) and body weights (F) of flies on a strawberry diet supplemented with protein (light gray) or with lipid or sugar or with mixtures of lipid and sugar (dark gray). Data were analyzed using Student’s *t* test or ANOVA followed by Tukey *post hoc* tests or the Wilcoxon rank sum test or Kruskal-Wallis test followed by Dunn’s test with Bonferroni correction depending on the results of a Shapiro-Wilk normality test. Different capital letters indicate significant statistical difference at a *P* value of <0.01. "Dead" indicates that no adult fly survived that treatment. Values equaling zero were excluded from statistical analysis. (The total numbers of the replicates are shown in Table S2.) (G) Relative levels of gene expression of *InR* in CR (red) and GF (green) larvae that were grown on a strawberry diet for 2 days and 5 days after egg deposition. Relative Δ*_CT_*^*InR*^/Δ_*CT*_^*Rpl32*^ ratios are represented. Data represent means ± SEM. *n* = 7 to 9. NS, not statistically significant. *, *P* < 0.05 for Wilcoxon rank sum test. Download FIG S5, TIF file, 1.4 MB. Copyright © 2018 Bing et al. 2018 Bing et al. This content is distributed under the terms of the Creative Commons Attribution 4.0 International license.

### Microbiotas serve as food for *D. suzukii* development.

We hypothesized that microbes could sustain larval development either by aiding in the digestion of food (40) or by serving as a direct nutrient source for the fly (44). To test these hypotheses, we supplemented the fruit-based fly diets with heat-killed *A. persici* or *S. cerevisiae* and monitored the development of CR and GF flies. We found that a one-time introduction of a very low dose of heat-killed microbes (e.g., optical density at 600 nm [OD_600_] of 0.1 = ~5 × 10^6^ *Acetobacter* cells or ~1.5 × 10^5^ *Saccharomyces* cells) did not rescue development of flies on a strawberry-based diet (Fig. 5C). However, increasing the quantity of the supplement of heat-killed microbes provided with the food rescued fly development in a dose-dependent manner (Fig. 5C; see also Fig. S5C). The amount of *Saccharomyces* cells needed to rescue development (~1.5 × 10^6^ cells) was less than the amount of *Acetobacter* cells needed (~5 × 10^8^ cells), but their biomass was heavier (~0.31 mg dry cell weight of *Saccharomyces* versus ~0.24 mg dry cell weight of *Acetobacter*), suggesting that different microbes provide nutrients in different quantities, possibly as a function of their biomass. Although the developmental rates were lower in flies fed heat-killed microbes than in CR flies, the size of the rescued adults was similar to that of the flies associated with live microbes (Fig. 5D; see also Fig. S5D). Additionally, when the fruit-based diet was complemented with a high quantity of autoclaved yeast (5% [wt/wt] yeast), GF developed with no noticeable lagging compared to CR flies (Fig. 5E and F; see also Fig. S5E and F). On the blueberry-based diet, only one adult fly emerged when the diet was supplemented with a low dose of heat-killed microbes (OD_600_ = 0.1), and that fly developed much more slowly than the live-microbe-associated controls (Fig. S5C and D). No consistent correlation between dose and emergence time was detectable for flies associated with live microbes on either blueberry- or strawberry-based diets (Fig. 5C; see also Fig. S5C), suggesting that microbe-derived nutrients are not limiting when *D. suzukii* is associated with live bacteria. Taken together, though our experiments cannot strictly rule out the possibility that specific microbial activities support development, our results support the hypothesis that microbes are taken in by *D. suzukii* as food and support fly development by providing key nutrients.

### *D. suzukii* larvae require protein for development on fruit-based diets.

Given the knowledge that microbes serving as food allow the development of *D. suzukii* on fruit-based diets, we next wanted to identify what key nutrients these microbes provide to *D. suzukii*. For this purpose, we supplemented blueberry- and strawberry-based diets with four different categories of nutrients—protein, lipid, carbohydrate (or, specifically, sugar), and a cocktail of vitamins and minerals—and monitored the development of GF flies on these diets. Protein (2% bovine serum albumin or casein) supplementation was the only condition that rescued larval development, and adult flies emerging from that supplemented diet had body sizes similar to those of CR flies but a lower development rate (Fig. 5E and F; see also Fig. S5E and F). Diets that included a mixture of all of the categories of nutrients did not rescue development significantly better than supplementation of protein alone (Fig. 5E and F; see also Fig. S5E and F), suggesting that protein is the key nutrient lacking in fruit-based diets for *D. suzukii*.

### Flies starve in the absence of microbiota on fruit-based diets.

In *D. melanogaster*, the insulin signaling pathway controls larval growth and development, and protein starvation abolishes insulin signaling, activating FOXO-dependent starvation responses (45). One landmark of FOXO activation is the transcriptional upregulation of the insulin receptor (InR) to prime the host to respond to insulin when it again becomes available (46). We hypothesized that if protein were limiting in fruit-based diets, GF larvae would show signs of protein starvation whereas CR flies would not. We therefore monitored *InR* gene expression by reverse transcription-quantitative PCR (RT-qPCR) in GF and CR *D. suzukii* larvae and found that *InR* was significantly upregulated in GF larvae compared to CR larvae after 5 days of growth on fruit-based diets (Fig. 5G; see also Fig. S5G). These results demonstrate that *D. suzukii* suffers from protein starvation on fruit-based diets in the absence of microbiota.

## DISCUSSION

In this study, we provide new data that further our understanding of the diverse roles of microbes in *D. suzukii* and in *Drosophila* species in general. On the basis of our collective experimental evidence, we present a model that suggests that the microbes associated with *D. suzukii* can be either deleterious or beneficial, depending on the nutritional environment (Fig. 5H). For flies on a protein-rich, nutrient-sufficient diet, the microbiota is not required for larval development, and the presence of the microbes limits both body size and life span. In contrast, for flies on diets corresponding to their natural niche, i.e., fruit-based diets, the microbiota is essential for fly development. We further demonstrate that protein is a limiting factor with a fruit-based diet and that microbes serve as a key source of protein to rescue starvation-dependent developmental arrest in *D. suzukii*. Taken together, our findings are indicative of the complex interaction between a developing host and its microbiome and point to the importance of evaluating the role of the microbiota in multiple physiological situations.

### *D. suzukii* does not have a core microbiome.

Our study, along with several other reports on the bacterial community of *D. suzukii* (31–33), attempted to identify a core microbiota for *D. suzukii*. However, given the considerable amount of variation found among such investigations, we could not find a strictly defined core of bacterial species that are always associated with *D. suzukii*. Although associations with some higher taxonomic categories (such as *Enterobacteriaceae*, *Acetobacteraceae*, and *Staphylococcaceae*) were repeatedly found in multiple studies, different populations harbored differing genera and species. For instance, among the members of the family *Enterobacteriaceae*, the dominant bacteria were *Tatumella* in a study by Chandler et al. (31), *Enterobacter* and *Lonsdalea* in a study by Martinez-Sañudo et al. (32), and *Dickeya* in our present study (Fig. 2C). *D. suzukii* was reported to harbor different *Acetobacteraceae* species in different studies (31–33), but *Acetobacteraceae* species were not prevalent in our own sequencing results. A similar situation has been documented in *D. melanogaster*, where *Acetobacteraceae* species were present in one study (38) but absent in another (16). The observed variation in microbial communities among different microbiotas in sequencing studies may be explained by differences in sampling location and time. Differences in experimental procedures may also introduce bias in microbiota sequencing analysis (47–49). Taken together, our results suggest that *D. suzukii* is constantly associated with microbes of few taxa, but it is likely that these microbes are constantly ingested from the environment and could be considered “dietary microbes.”

We found that bacterial communities varied significantly with respect to sampling location and collecting time. However, as the *D. suzukii* flies used in this study were collected from only two field locations, this may limit the scope of conclusions about the variability of associated microbes, and additional sampling might reveal new patterns or some level of microbial community structure. However, the significant spatial and temporal variation of microbially diverse populations in *Drosophila* species, including *D. suzukii* (18, 36, 38, 50), suggests that *Drosophila* species generally do not have a consistent pattern of microbiota in the wild due to constant changes in habitat as well as other factors. As with *D. melanogaster* (18), we found a significant difference between wild and laboratory populations of *D. suzukii* with respect to bacterial communities. This contrast shows that the pattern of microbial populations present in *Drosophila* is unlike that seen with plant phloem-sucking insects (such as whiteflies), in which the insects representing laboratory samples and ﬁeld samples harbor similar microbes (51). One explanation for this phenomenon is based on differences in the degree of association between a host and its microbiota, with *Drosophila* flies possibly relying more than other insects on their dietary microbes to replenish their microbiota, as was suggested by Blum et al. (52). A host-microbiota system that relies on dietary microbes would be more variable and less constantly maintained, depending on the host habitat (50), and in that case, diet would have a major influence on the microbial composition of the host insect (53–56). Our results, along with those from a recent study (33), suggest such a model for *D. suzukii*. Our laboratory populations show very restricted bacterial diversity, suggesting that laboratory conditions strongly affect the diversity of the microbiota. This could simply be a consequence of the nutrients and antimicrobials found in standard fly diets (57). Interestingly, laboratory stocks still maintained unique bacterial structures, despite being raised for 2 years under laboratory conditions. This may be explained by the fact that some microbes can stably colonize the *Drosophila* gut (58). Alternatively, it could be a consequence of the method of rearing flies in the laboratory, where bacteria are transmitted among flies during line maintenance.

The major bacteria identified in our sequencing data belonged to the families *Comamonadaceae*, *Enterococcaceae*, *Lactobacillaceae*, *Leuconostocaceae*, and *Pseudomonadaceae*, which were also detected previously in *D. suzukii* (32, 33) and other *Drosophila* species (16, 18, 37–39). The fungi/yeasts isolated in this study were *Candida*, *Hanseniaspora*, *Pichia*, and *Saccharomycetes* sp., which are known to be prevalent in *D. suzukii* in the field, as well as in other *Drosophila* species (30, 40, 59). *H. uvarum* was the most prevalent microbe in wild *D. suzukii* according to field investigations (30) and initiates a strong olfactory attraction for *D. suzukii* when added to blueberry (40). Female flies tend to feed more on yeast-associated blueberries in order to obtain nutrients. When scientists exposed ﬂies to a mixture of *H. uvarum* and insecticide, flies laid fewer eggs and exhibited a higher death rate than were seen with insecticide exposure alone (35). The interactions between *D. suzukii* and *H. uvarum* would be interesting to explore and might guide development of new methods for pest control.

### The impact of *D. suzukii* microbiota depends on diet.

Despite variation in their microbiota, we found that conventionally reared (CR) *D. suzukii* consistently performed worse than or similarly to germ-free (GF) flies in development and fitness experiments, when provided a nutrient-rich diet. The similar developmental rates and smaller adult body sizes of CR flies revealed a nonessential or even deleterious role of microbes in the development of *D. suzukii*. In contrast, the development of CR *D. melanogaster* larvae was previously shown to be similar to or slightly faster than that seen with GF flies on a nutrient-rich diet (16, 17, 60), suggesting that *D. melanogaster* is better adapted to live in a microbe-rich environment than *D. suzukii*. This phenomenon could be explained by the different ecologic niches of the two species; *D. suzukii* females deposit eggs in ripening and ripe fruit, and their larvae consequently may be less extensively exposed to certain bacteria or fungi than *D. melanogaster* larvae, which develop in rotten fruits associated with multiple species of microbes (29, 61). In agreement with this possibility, we found that *D. suzukii* GF flies have a significantly longer life span than CR flies. Recent studies have demonstrated that during aging, increases in bacterial density significantly impact several aspects of the host physiology of *D. melanogaster*, including the immune response, tissue homeostasis, gut physiology, and metabolism (20, 22, 24, 62, 63). Additionally, it is known that the microbiotas of multiple laboratory stocks alter *D. melanogaster* physiology and life span differently (20, 23, 64, 65). In our study, host life span was more affected by associated microbes in *D. suzukii* than in *D. melanogaster*. These results might therefore suggest that *D. suzukii* flies may be more susceptible to the stress induced by microbes during aging. Alternatively, because the microbiota associated with flies under laboratory conditions is only a reduced and selected version of their microbiota in the wild, it is possible that the conclusions that we draw by comparing *D. suzukii* and *D. melanogaster* are confounded by the specific association of the stocks with their subset of bacteria. Future studies should compare the effects of multiple controlled bacterial communities on host physiology to generalize the role of the microbiota in the wild and to better describe evolutionary relationships. Taking the data together, we can conclude that the microbiota of *D. suzukii* on nutritionally rich diets is, if anything, slightly deleterious to host physiology.

Interestingly, the opposite phenotype was observed when the same experiments were done using fruit-based diets, the natural source of food for *D. suzukii* larvae. We found that GF flies were unable to develop on blueberry- and strawberry-based diets and had a significant developmental delay on a raspberry-based diet. Interestingly, raspberries have almost double the amount of protein (1.2 g/100 g) that blueberries or strawberries have (0.7 g/100 g) (42), suggesting that protein might be more limiting in these fruits. Under these conditions, flies stopped developing at the second instar stage and reassociation with the microbiota of CR flies successfully rescued this developmental defect, indicating the importance of microbes to hosts feeding on fruit, a poor source of proteins. Similar phenotypes were observed in *D. melanogaster* as well, which is consistent with the previous reports noting that GF *D. melanogaster* grows slower in nutrient-poor environments (16, 17). The beneficial effect of the combination of microbes and fruits in our study supports the theory that microbes have the important role of supplementing nutrients to hosts living on nutrient-poor or unbalanced diets (66).

Our results further show that adults can develop from larvae monoassociated with either bacteria or fungi and fed on both strawberry- and blueberry-based diets, suggesting that the presence of microbes, rather than one specific form of microbial activity, is required to sustain development. The rescuing levels of efficiency differed among the studied microbes, but only a few of the tested bacterial species failed to aid in the complete development of the larvae. The variations in rescue efficiency of different microbes might be explained by the complex characteristics of various microbes (17, 44, 67, 68) or simply by their ability to grow to different levels on different fruit-based diets. For instance, *E. faecalis* titers have been shown to decline in *D. melanogaster* larvae (16). Since *E. faecalis* cannot colonize the fly gut in larvae, *E. faecalis* growth might be insufficient to compensate for the protein-poor diet. Quantitative studies (bacterial load assays) are required in order to assess microbial numbers and allow clear comparisons between bacterial species.

It is noteworthy that *H. uvarum* and *S. cerevisiae* can fully rescue fly development, implying that these yeasts are important contributors to the development of CR flies living on fruits. *Drosophila*-yeast associations are common in nature (69), and yeast can inﬂuence the survival, development time, and adult body weight of *D. melanogaster* (67). *H. uvarum* is widely associated with wild *D. suzukii* flies and can strongly attract flies for feeding (30, 35, 40). It is therefore possible that *D. suzukii* adults deposit these yeasts when they lay their eggs, thus providing their progeny with beneficial microbes in an otherwise protein-deficient environment.

### Flies eat microbes as food.

The fact that adding both heat-killed bacteria and fungi successfully rescued fly development on strawberry and blueberry agrees with the idea that microbes serve as food for *Drosophila* (37, 44, 70). Another model, which states that microbes help digest food by transferring unavailable nutrients to hosts in an available form, fits insects such termites and the tsetse fly (71, 72) but may not be suitable for all insects. In addition, we demonstrate that protein is the limiting nutrient needed by *D. suzukii* to develop on fruits. Several lines of evidence suggest the following: first, the beneficial impact of the microbiota is seen in flies fed with protein-limited food such as fruit-based diets; second, protein is a common nutrient present in all microbes; and third, adding protein alone to fruits was sufficient to rescue fly development. Finally, our results demonstrate that GF flies raised on fruit-based diets show signs of protein starvation. These results are in agreement with multiple studies showing that proteins are key limiting nutrients for larval growth in *D. melanogaster* (8, 16, 73). Collectively, these data suggest that *D. suzukii* mainly acquires protein from microbes growing within the fruit. However, the addition of protein alone significantly rescued development only, and additional physiological parameters were not fully rescued to the levels seen with CR flies, suggesting that other nutrients are needed for fly development. Recently, we found that vitamin B_1_ provided by microbes is important for the development of *D. melanogaster* (91). However, mixing protein and multiple vitamins or mixing protein, lipid, and vitamins did not altogether improve the rescue of GF flies on fruit-based diets. It is possible that specific ratios of nutrients are needed to fully rescue all of the physiological parameters. Accurate identification of the precise nutrients required for *D. suzukii* development would be an interesting aim for further studies.

In conclusion, *D. suzukii* harbors a diverse array of microbes, which change significantly due to location and time. Naturally living in a relatively nutrient-poor environment, *D. suzukii* has evolved to eat microbes as food to sustain its development under nutrient-poor conditions. However, as a compromise, one disadvantage of that relationship is that the presence of the microbes may impose stress on *D. suzukii* when nutrients are fully provided by the food. The model in our study reveals the importance of microbes for *D. suzukii* development and the complicated interaction between *Drosophila* flies and their microbial communities.

## MATERIALS AND METHODS

### Fly strains and rearing.

*D. suzukii* Cali and NY and *D. melanogaster* Canton S fly populations were kept at between 22°C and 25°C with a 12-h/12-h light/dark cycle. Flies were maintained in plastic *Drosophila* vials and round-bottom bottles (VWR International, Radnor, PA) and fed our optimized sucrose food (X. L. Bing, J. Winkler, J. Gerlach, G. Loeb, N. Buchon, submitted for publication). The optimized food contains 4% (wt/vol) sucrose (VWR, Radnor, PA), 5% brewer’s yeast (MP Biomedicals, Santa Ana, CA), 6% yellow cornmeal (Aunt Jemima, Chicago, IL), 0.7% fly agar (MoorAgar Inc., Rocklin, CA), 0.13% (wt/vol) methyl paraben (Moldex; Sigma-Aldrich Corp., St. Louis, MO), and 0.498% (vol/vol) phosphoric acid (Sigma-Aldrich Corp., St. Louis, MO). The Cali population is a homogenous population obtained from the Chiu laboratory at University of California, Davis. The NY population was collected in 2015 from farms in New York state and maintained in the laboratory. Before extraction of DNA for the microbiota assay was performed, all flies were reared on the optimized food for at least 10 generations (see Table S1 in the supplemental material).

### Collection of wild *D. suzukii* adults.

*D. suzukii* wild adults were collected from two fruit farms (LO and RPE) near Geneva, NY, by attracting flies into traps using crushed fresh fruit (raspberry and blueberry) as attractive bait. The traps were constructed from opaque plastic containers (Thermo Fisher Scientific, Waltham, MA) (1,892.74 ml) ringed with 44 holes (3.175-mm diameter), enabling flies to enter. Flies were prevented from gaining access to the fruit by enclosing the bait with a fine mesh fabric. Flies from each farm were collected in early September and early November 2016 (Table S1). The LO farm grew mostly sweet cherries, while the RPE farm grew mostly blueberries. After collection, the flies were anesthetized with ice, sorted by sex, submerged in sterile distilled water, surface sterilized in 70% ethanol three times, rinsed in sterile distilled water, and finally stored in 100% ethanol until DNA extraction was performed. The purpose of this procedure was to minimize the presence of bacteria on the fly surface.

### Bacterial community characterization.

To identify the microbial community in flies, 10 individual flies collected from wild and laboratory populations were pooled as one replicate. Each treatment had three replicates. The bacterial DNA was extracted and purified from the samples using a MasterPure Gram-positive DNA purification kit (Epicentre, Madison, WI) according to the instructions provided by the manufacturer. The V3-V4 region of 16S rRNA gene was amplified by PCR using the 341F and 805R primers, as previously described (74). The resulting amplicons were purified using AMPure XP beads (Beckman Coulter, Inc., Indianapolis, IN) and then pooled and sequenced on an Illumina MiSeq platform (Genomics Facility, Cornell University, NY) using a paired-end technique (2 × 300 bp).

The sequenced paired Illumina reads were merged with PANDAseq version 2.11 (75). High-quality reads were processed for quality filtering, chimera removal, and operational taxonomic unit (OTU) picking using the usearch61 algorithm, which incorporates a chimera check, and other quality filtering pipelines (76, 77) implemented in QIIME version 1.91 (78). Chimeras were identified using *de novo* and reference-based chimera checking by UCHIME (77) within the pipeline. OTUs were clustered at 97% identity. Taxonomy was assigned with UCLUST (76) using the Greengenes reference database version 13.8 (79). Filtered representative sequences were aligned using PyNAST (78), and a phylogenetic tree was constructed using FastTree (80). The 16S rDNA gene sequencing approach generated 3,311,535 reads with an average of 91,987 reads per sample and yielded 44,518 operating taxonomic units (OTUs). All communities were rareﬁed to 15,500 reads per sample for diversity analysis. Alpha diversity data measuring levels of observed OTUs and phylogenetic diversity were calculated in QIIME and compared with Student’s *t* test or multiple analysis of variance (ANOVA) data. For beta diversity data, the unweighted and weighted UniFrac distances (81) between communities were calculated with the R package “phyloseq” (82) and analyzed using Adonis (also referred to as "permutational multivariate analysis of variance" [PERMANOVA]) (83). To better represent variations within each factor, we analyzed the testing factor and consolidated the other factors. Diversity visualization was realized with the R package “ggplot2” (84).

### Isolation and identification of culturable laboratory microbes.

Two female and two male *D. suzukii* flies from each laboratory population were randomly chosen and washed with distilled water and ethanol as described above, homogenized in a centrifuge tube containing 500 µl PBS and one metal bead using a FastPrep-24 instrument (MP Biomedicals, Santa Ana, CA), and plated on De Man, Rogosa, and Sharpe (MRS) (Himedia Laboratories, West Chester, PA), lysogeny broth (LB), and Sabouraud dextrose agar plus yeast extract (SDAY) plates using a wasp 2 spiral plater (Microbiology International, Bethesda, MD). Following incubation at 29°C, colonies growing on the plates were checked after a minimum of 24 h. On the basis of the morphology seen, colonies were picked and further purified on corresponding plates until single colonies were separated on agar plates.

Microbial DNAs were extracted from single colonies using an alkaline heat-extracted method and a TaKaRa Fungal rDNA (ITS1) PCR Fast kit (TaKaRa, Kusatsu, Shiga, Japan). Microbes were identified by sequencing the amplifications of either the bacterial 16S rRNA gene or the fungal internal transcribed spacer (ITS) region. The bacterial 16S rRNA gene was amplified using universal primers 27F, 530F, and 1495R (92). The fungal ITS region was amplified with universal primers ITS4 and ITS5 (93). PCR was performed using 2× GoTaq green master mix (Promega, Madison, WI) and an S1000 thermocycler (Bio-Rad, Hercules, CA). The PCR procedure used has an initial step of 95°C for 3 min, followed by 35 cycles of 95°C for 30 s, 55°C for 30 s, and 72°C for 45 s and a final step of 72°C for 10 min. Amplified DNA products were visualized by agarose gel electrophoresis and then purified using a Monarch PCR and DNA cleanup kit (New England Biolabs, Ipswich, MA). The purified PCR products were sequenced on ABI 3730xl DNA analyzers (Applied Biosystems, Waltham, MA). Sequencing results were checked by blastn in the NCBI nr database (http://blast.ncbi.nlm.nih.gov/Blast.cgi). The top blast hit was assigned to the isolated microbe.

### Oral infection.

Adult male flies (4 to 8 days old) were incubated for 3 h at room temperature in an empty vial before being transferred to a vial containing microbial solution. The infection solution was obtained by mixing equal volumes of concentrated pellet from a suspended culture of bacteria or fungi (OD_600_ = 10 or 200) and of a solution of 5% sucrose (1:1 [vol/vol]). This solution was deposited on a round filter disk that completely covered the surface of the sucrose-yeast food. Flies were initially incubated for 1 day on the contaminated filter before they were transferred to vials with fresh food. For the unchallenged controls, microbe solutions were replaced with PBS. All experiments were performed at 25°C on the optimized sucrose food. Flies were moved onto fresh food every 2 days and counted every day to monitor survival for 1 week. Survival curves represent at least three independent repeats of 20 individuals (≥60 flies tested in total).

### Generation of germ-free flies.

We referred to the method previously described (85) to generate germ-free (GF) *D. melanogaster* flies. To produce GF *D. suzukii* flies, we followed the same protocol with modifications. In the modified protocol, 4-to-8-day-old flies were allowed to lay eggs on a plate of live baker’s yeast for 4 to 6 h. The eggs were then collected on mesh baskets by rinsing the yeast with distilled water and then washing twice with PBS, once with 70% ethanol, once with 0.6% hypochlorite (equivalent to 10% Clorox bleach), and 3 times with sterile water. The eggs were then aseptically transferred to autoclaved food in a biosafety cabinet. Vials suspected of contamination were inspected by plating crushed flies suspended in PBS on MRS and SDAY plates. For conventionally reared (CR) flies, embryos from the same collection were transferred to autoclaved food before washing with ethanol was performed. To prepare microbiota-associated GF (GF+ Micro) flies, GF flies were associated with microbes obtained from CR flies, which have microbes by default. In brief, microbes of 2 female and 2 male CR flies were released in 500 µl PBS solution after homogenization, and 50 µl of this solution was added to each vial.

### Microbe association of GF flies.

For generating defined microbe-associated flies, we first found representatives of microbes identified from laboratory-reared and wild *D. suzukii* as well as some microbes from *D. melanogaster*. In detail, *Acetobacter persici*, *Enterococcus faecalis*, *Lactococcus lactis*, *Staphylococcus epidermidis*, *Hanseniaspora uvarum*, *Pichia kudriavzevii*, *Rhodotorula mucilaginosa*, and *Saccharomycetes* sp. were isolated from laboratory flies (Fig. 2). *Delftia tsuruhatensis* was isolated from wild *D. suzukii* (Bing et al., submitted). *A. pomorum*, *A. tropicalis*, *Lactobacillus brevis*, and *L. plantarum* were isolated from laboratory *D. melanogaster* flies. *D. acidovorans* was obtained from the Cornell Department of Microbiology laboratory teaching collection. *Leuconostoc mesenteroides* was obtained from the USDA ARS Culture Collection (NRRL) (https://nrrl.ncaur.usda.gov/).

Bacteria or fungi were added to the food surface immediately after transfer of germ-free eggs. The microbes used in this process were cultured overnight at 29°C and centrifuged at 3,000 × *g* for 10 min and then resuspended in PBS at a certain optical density (OD_600_ = 0.1, 1, or 10). A 50-μl volume of resuspended cells was added to each vial. For treatment of dual associations, the two microbial species were added in equal parts. The day of microbe addition to the food surface was marked “day 0” for all studies. *L. brevis* and *L. plantarum* were cultured overnight in MRS broth at 29°C without shaking. Heat-killed microbes were prepared by autoclaving at 121°C for 25 min.

### Development time assay.

After eggs were added to different foods, the dates of emergence of individual flies were recorded daily for analysis. Conditions under which no flies developed were excluded from statistical comparisons.

### Quantification of fly larva length, adult weight, and wing length.

Larvae were picked up from fruit vials and were transferred into a glass spot plate containing water. The larvae were then heat-killed by microwaving for 10 s. The bodies of the larvae straightened out after microwaving and were arranged and photographed on a slide under an Amscope MU300 microscope (Amscope, Irvine, CA). A ruler was set beside the larvae as a reference. Newly emerged flies were collected and weighed on a Mettler Toledo XS3DU microbalance (Mettler Toledo, Greifensee, Switzerland). Flies that emerged from the sucrose food were weighed in pairs, and the weight of each adult was calculated afterward. Flies from the microbe rescue and nutrition rescue experiments were weighed individually. Quantification of wing length was performed as previously described (86). In brief, one wing from each emerged fly was removed and wings from the same treatment were positioned under a coverslip on a glass slide. The pictures were taken using ×10 magnification and a Zeiss Axio Observer Z1 microscope and measured with ZEN 2012 (blue edition) (Carl Zeiss, Inc., Oberkochen, Germany). Wing length was measured as the linear distance from the intersection of the anterior cross vein to the wing margin at the distal end of the third longitudinal vein. Both female and male wings were cut and measured.

### Longevity assay.

Newly emerged CR and GF flies were transferred to sterile sucrose diet vials. Flies were moved to new sterile food in a germ-free biosafety cabinet every 2 to 3 days and checked daily until every fly had died. The date of death of each fly was recorded for longevity calculation. For each experiment, at least 3 vials were set up for each diet, and this experiment was repeated two times (*n* > 120).

### Fecundity assay.

Two newly emerged female and male GF and CR flies were transferred to a vial with sterile sucrose food. Flies were then allowed to lay eggs for 7 days. After removal of the parental generation, the emerged adult offspring were counted for each vial. The average number of adult offspring per female was calculated as an index of fly fecundity. Each condition was measured in at least three biological repeats, each consisting of >3 vials. Each single vial represented one sample. All data were combined for statistical comparisons.

### Nutrition supplement experiments.

The fruit diets were made by crushing fresh blueberries and strawberries (from Wegmans and BJ’s Wholesale Club) with a homogenizer followed by supplementation with 2% fly agar (MoorAgar Inc., Rocklin, CA). After autoclaving, the fruits were transferred into autoclaved glass vials in a sterilized cabinet. To identify the nutrient(s) responsible for recovery of fly development on GF fruits, protein (bovine serum albumin [BSA] and casein), lipid (coconut oil and lard), sugar (glucose and sucrose), multiple vitamins and minerals, and yeast were added separately or in combination to the fruit food. The amounts of protein and lipid were calculated based on their proportions in yeast. Except for the vitamins, all nutrients were added into fruits before autoclaving. Details of the dosage and composition of each nutrient are listed in Table S2. The date of emergence and fly weight were recorded by the use of the methods described above.

### RT-qPCR.

Total RNA was extracted from pools of ~20 larvae per sample point using TRIzol (Thermo Fisher Scientific, Waltham, MA). RNA was reverse transcribed using a qScript cDNA synthesis kit (Quantabio, Beverly, MA), and qPCR was performed using SsoAdvanced Universal SYBR green Supermix (Bio-Rad, Hercules, CA) and a CFX96 real-time PCR detection system. For samples that had undetectable levels of mRNA (N/A), the maximum number of cycles (number of quantification cycles [*Cq*] = 40) was given to replace N/A. The expression data represent the ratio or relative ratio of mRNA levels of the target gene (*InR*; DS10_00012616) to that of a reference gene (*RpL32* [also known as *rp49*]; DS10_00012899). The primer sequences used in this study were 5′-TGCCCACCGGATTCAAGAAG-3′ and 5′-TCTCTTGAGAACGCAGACGAC-3′ for *rpl32* and 5′-GCATCAAACGTGAAAGCGGT-3′ and 5′-ATCCAGCACATACAACGCGA-3′ for *InR*.

### Statistical analysis.

All analyses were performed using the statistical computing software environment R 3.3 (87). Developmental curves and survival and longevity data were analyzed via Cox proportional-hazards model test analysis using the “survival” package (88). The time of emergence, body weight, and wing size data were first analyzed by a Shapiro-Wilk normality test followed by parametric or nonparametric analysis. A Student’s *t* test or Wilcoxon rank sum test was used for comparisons of data from two treatments. Analysis of variance (ANOVA) with the Tukey *post hoc* test was conducted to compare data from multiple samples using the “multcomp” package (89). When the data did not follow a normal distribution, the Kruskal-Wallis test and Dunn’s test with Bonferroni correction from the “PMCMR” package were used for multiple comparisons (90).

### Accession number(s).

Sanger sequences of identified, culturable microbes have been deposited in the NCBI GenBank database under accession numbers MG201777 to MG201782 and MG250508 to MG250513. The raw Illumina sequencing data for the 16S rRNA sequences have been deposited in the SRA database under GenBank accession numbers SRR6130729 to SRR6130764.
