# Supplementary figures and images for: Nutrient-Dependent Impact of Microbes on Drosophila suzukii Development
Source: mBio. 2018 Mar 20;9(2):e02199-17. doi: 10.1128/mBio.02199-17 (PMC5874910; doi:10.1128/mBio.02199-17)

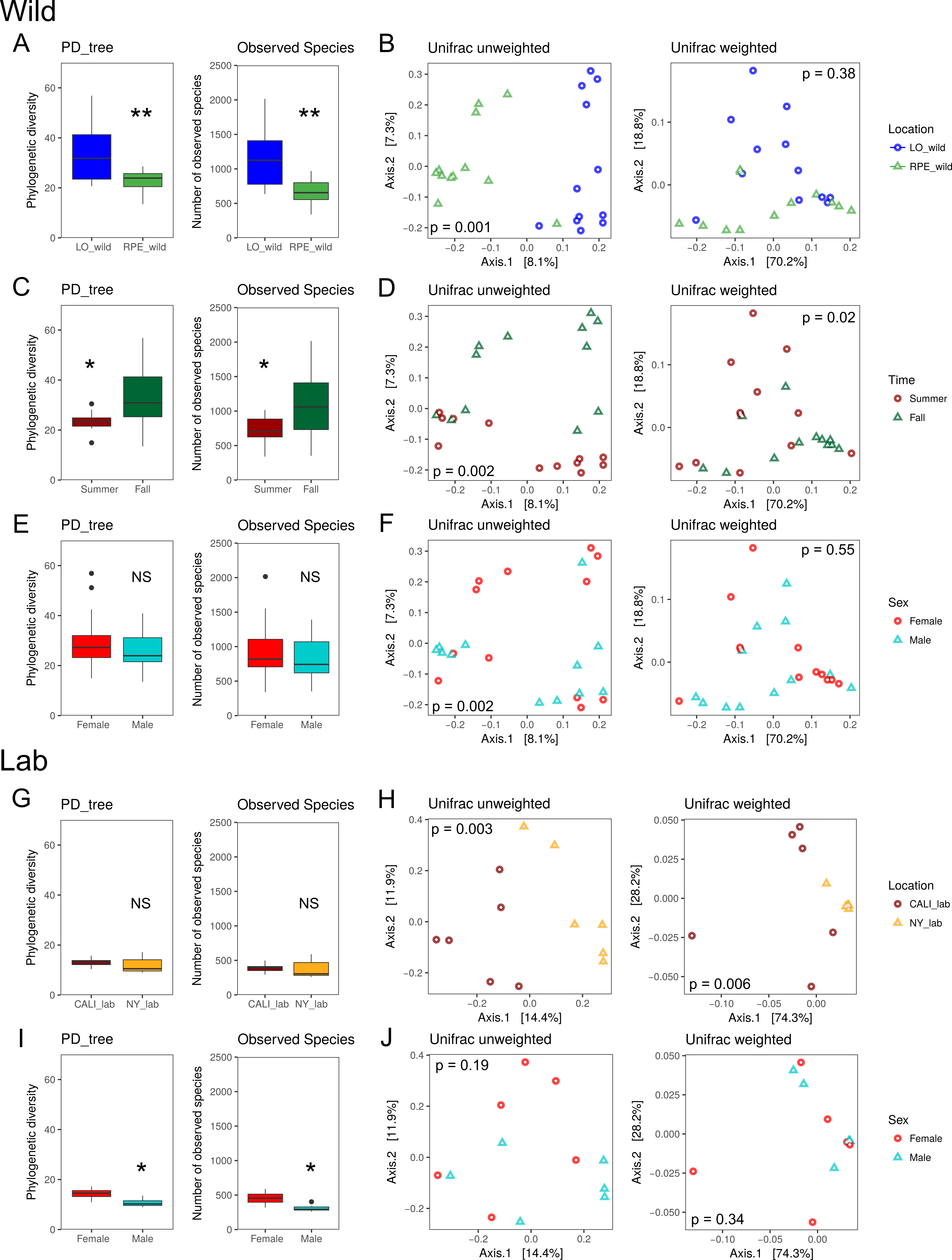

Supplement: FIG S1 [file mbo002183788sf1.tif]

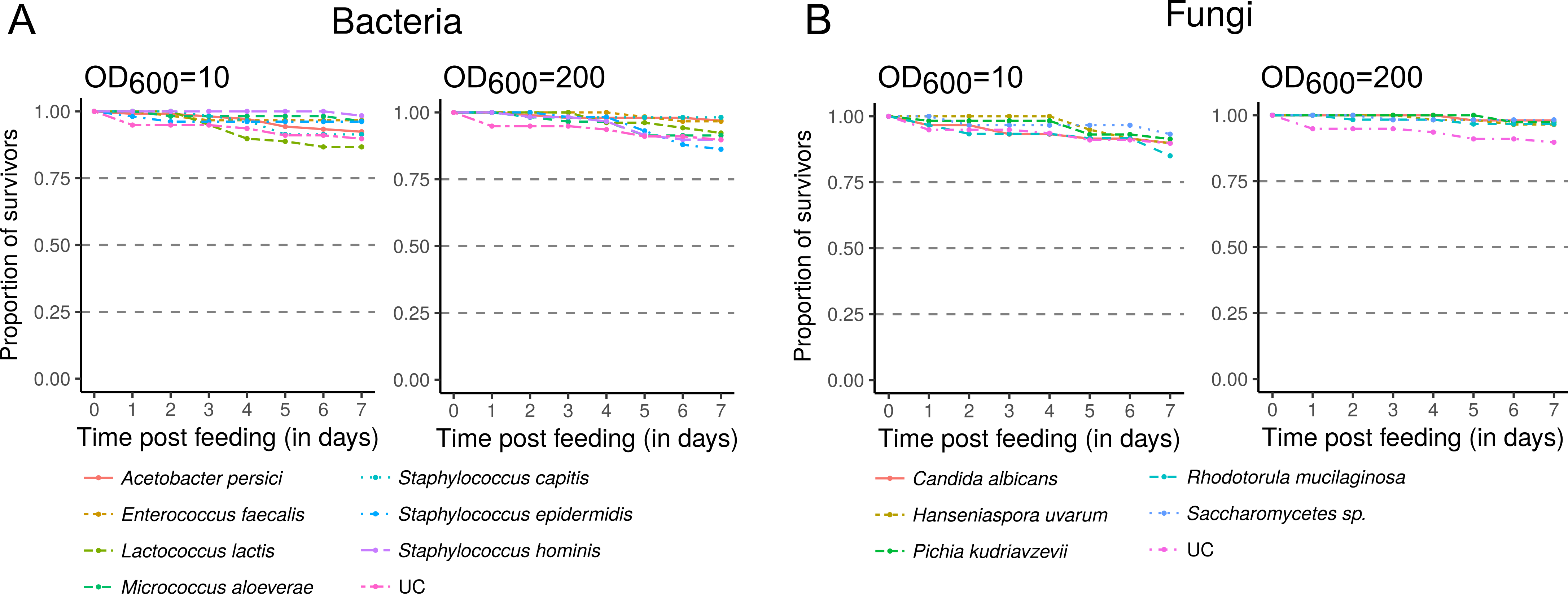

Supplement: FIG S2 [file mbo002183788sf2.tif]

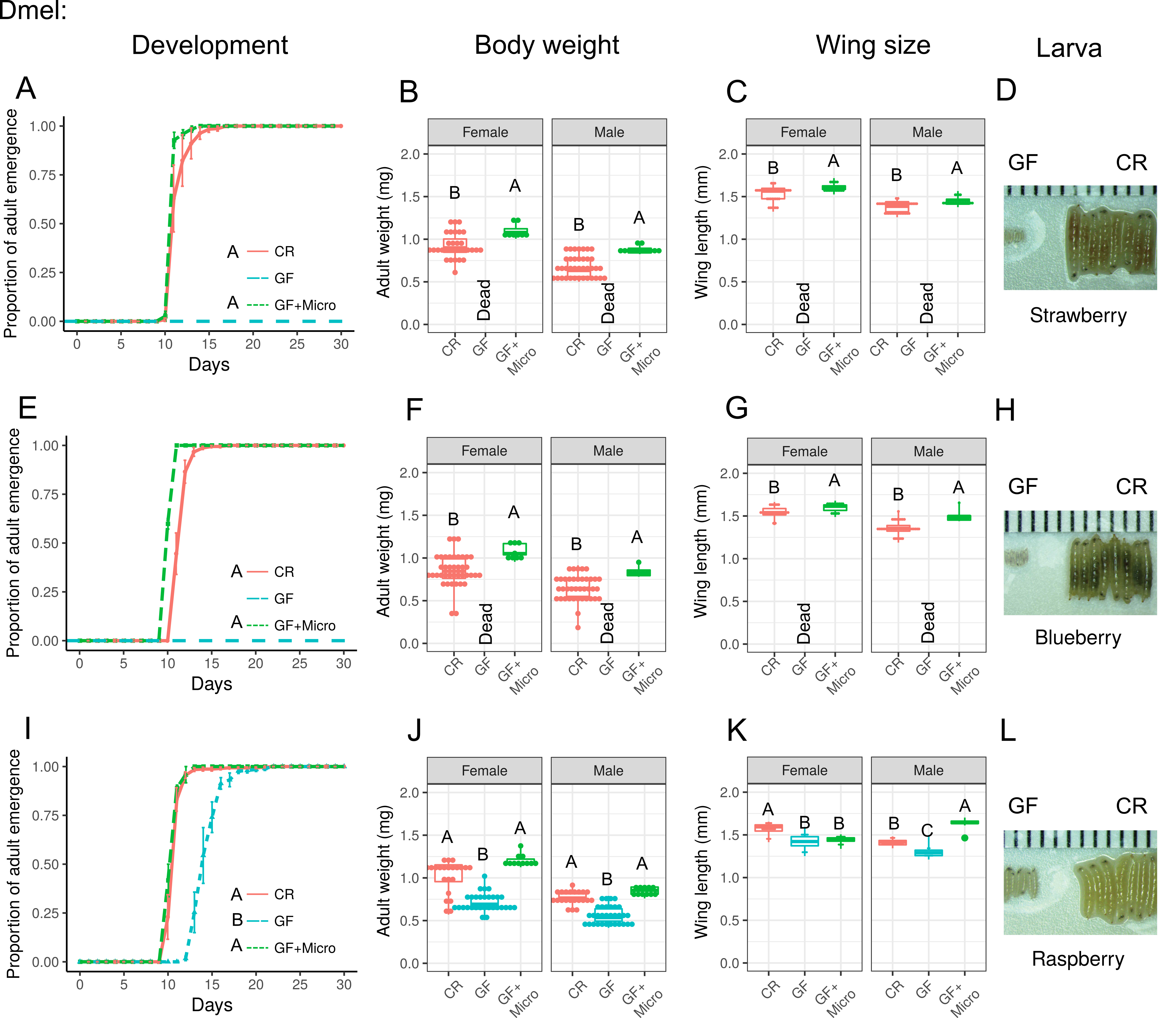

Supplement: FIG S3 [file mbo002183788sf3.tif]

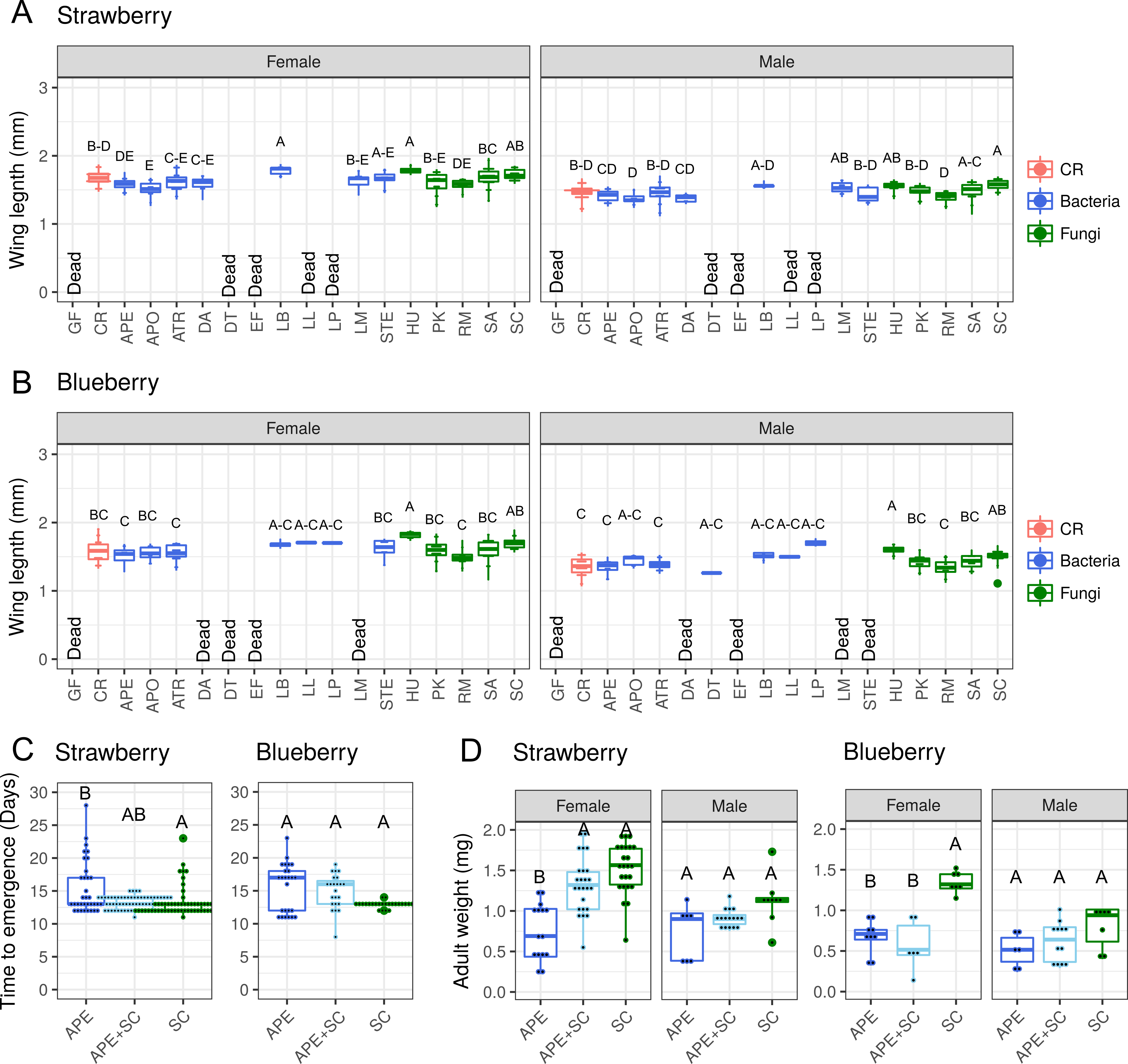

Supplement: FIG S4 [file mbo002183788sf4.tif]

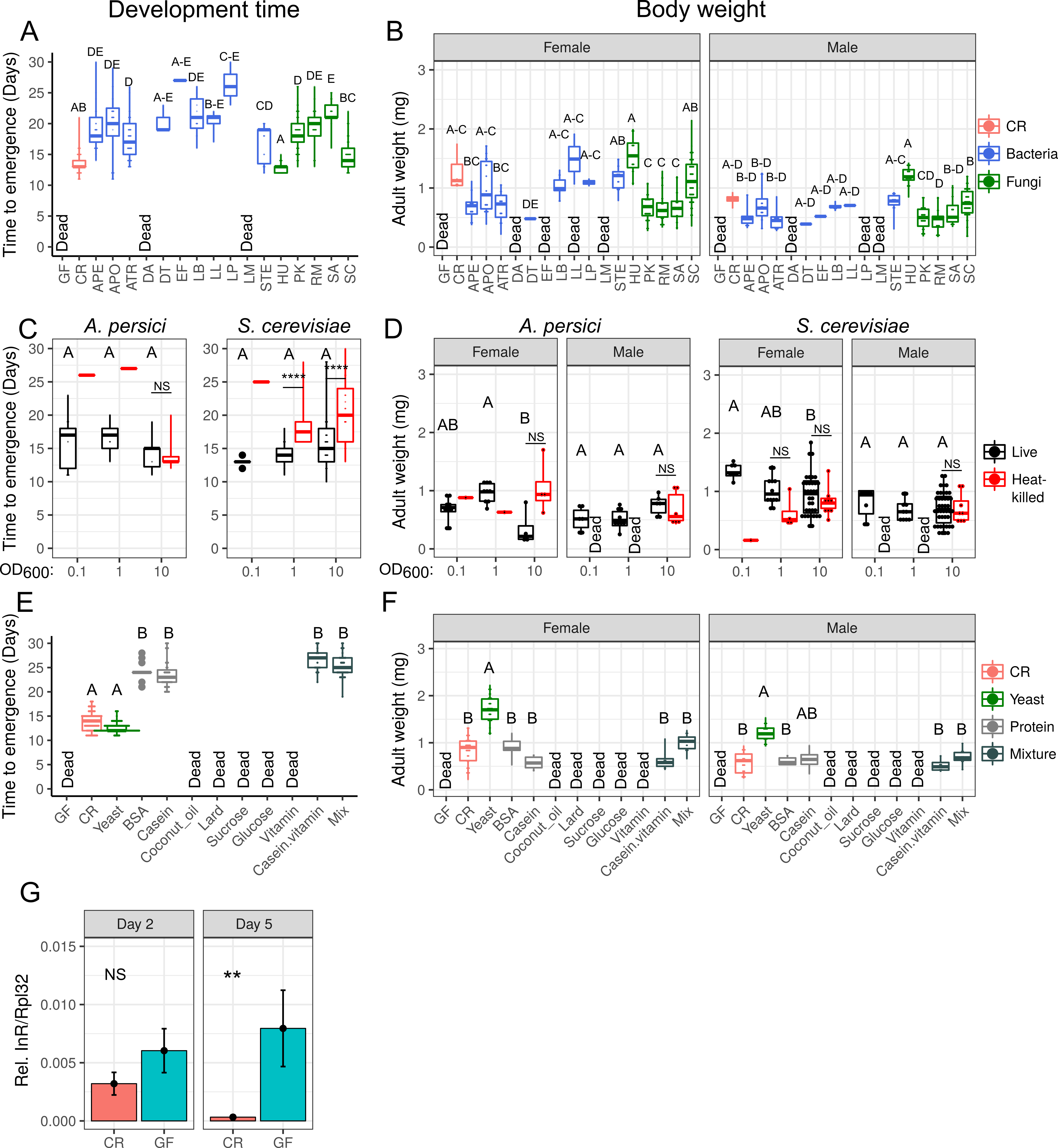

Supplement: FIG S5 [file mbo002183788sf5.tif]
